# Location and Effects of an Antitumoral Catechin on the Structural Properties of Phosphatidylethanolamine Membranes

**DOI:** 10.3390/molecules21070829

**Published:** 2016-06-24

**Authors:** Francisco Casado, José A. Teruel, Santiago Casado, Antonio Ortiz, José N. Rodríguez-López, Francisco J. Aranda

**Affiliations:** 1 Departamento de Bioquímica y Biología Molecular-A, Universidad de Murcia, Campus de Espinardo, Murcia E-30100, Spain; fcasadorojo@yahoo.es (F.C.); teruel@um.es (J.A.T.); ortizbq@um.es (A.O.); neptuno@um.es (J.N.R.-L.); 2 IMDEA-Nanoscience, Campus de Cantoblanco, Madrid E-28049, Spain; santiago.casado@imdea.org

**Keywords:** catechins, TMCG, phosphatidylethanolamine, DSC, X-ray diffraction, AFM, FT-IR, molecular dynamics

## Abstract

Green tea catechins exhibit high diversity of biological effects including antioncogenic properties, and there is enormous interest in their potential use in the treatment of a number of pathologies. It is recognized that the mechanism underlying the activity of catechins relay in part in processes related to the membrane, and many studies revealed that the ability of catechins to interact with lipids plays a probably necessary role in their mechanism of action. We present in this work the characterization of the interaction between an antitumoral synthetically modified catechin (3-*O*-(3,4,5-trimethoxybenzoyl)-(−)-catechin, TMCG) and dimiristoylphosphatidyl-ethanolamine (DMPE) membranes using an array of biophysical techniques which include differential scanning calorimetry, X-ray diffraction, infrared spectroscopy, atomic force microscopy, and molecular dynamics simulations. We found that TMCG incorporate into DMPE bilayers perturbing the thermotropic transition from the gel to the fluid state forming enriched domains which separated into different gel phases. TMCG does not influence the overall bilayer assembly of phosphatidylethanolamine systems but it manages to influence the interfacial region of the membrane and slightly decrease the interlamellar repeat distance of the bilayer. TMCG seems to be located in the interior of the phosphatidylethanolamine bilayer with the methoxy groups being in the deepest position and some portion of the molecule interacting with the water interface. We believe that the reported interactions are significant not only from the point of view of the known antitumoral effect of TMCG, but also might contribute to understanding the basic molecular mechanism of the biological effects of the catechins found at the membrane level.

## 1. Introduction

The presence of bioactive catechins in green tea has been implicated in the protection against various pathological conditions and the scientific interest in these polyphenols as therapeutic agents is rapidly increasing [1,2,3]. Catechins are potent antioxidant agents [4] and exhibit antifungal [5] and bactericidal activity [6,7]. Catechins have been found to have antiviral properties [8] and it has been reported that they are linked with protection against various diseases including cardiovascular, hepatic, renal, and neurological disorders [9].

**Figure 1:**
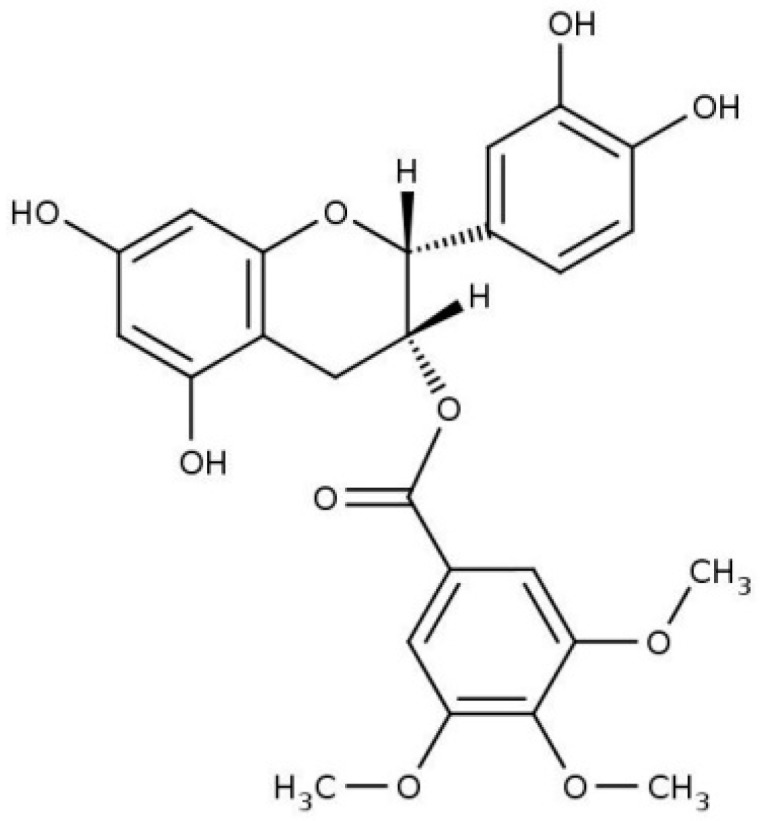
Structure of 3-*O*-(3,4,5-trimethoxybenzoyl)-(−)-catechin (TMCG).

There is evidence confirming that green tea catechins show strong anticancer effects [10,11] and this has led to intense research centered on the antioncogenic properties of these polyphenol compounds. The available information demonstrates that catechins inhibited growth of cancer cells in various types of cancer, such as lung cancer [12], prostate cancer [13], and others [14]. In this respect, one of us found earlier that the ester-bonded gallatecatechins, obtained from green tea, showed strong inhibition of dihydrofolate reductase at levels present in the organs and blood of tea drinkers [15,16]. In order to increase the bioavalilabity of catechins and their ability to incorporate into cellular membranes, a 3,4,5-trimethoybenzoyl analogue of catechin-3-gallate (TMCG, Figure 1) was synthesized and observed that it was able to inhibit the growth of melanoma cells [17].

Despite the high number of studies reporting biological effects of catechins, the underlying molecular mechanism is not completely elucidated. It is broadly recognized that the biological activity of catechins affects activities present in the membrane like signal transduction [18] and it has been shown to regulate various membrane proteins like ion channels [19] and receptors [20]. Damaging of membrane has been implicated in the destruction of bacterial biofilms [21], blockade of membrane fusion has been shown to interfere with cell virus entry [22] and alteration of membrane organization has been suggested to inhibit phenotypes related to cancer metastasis [23].

Considering all the above evidence, the interaction between catechins and membranes is emerging as an interesting field of investigation. It is known that catechins decrease membrane fluidity [24] and cause aggregation and leakage of contents from lipid vesicles [25,26]. Catechins partition into the lipid bilayers and perturb their structure [27] and it has been suggested that catechins may exert their effects on membrane function by a common bilayer mediated mechanism [19]. Despite that, the amphiphilic essence of TMCG advocates to the membrane as its theoretical place of action, there is limited information on the interaction between this antitumoral drug and membranes. We have previously observed that TMCG incorporates into and interacts with dipalmitoylphosphatidylcholine membranes, the data suggesting that the location of TMCG would allow it to reach tyrosinase at its membrane location and form its activated quinine methide product [28]. The examination of the influence of this catechin on the lipid component of membranes is very important to shed light on the mechanism of activation and transport of this compound and to investigate other possible activities. Phosphatidylethanolamines are crucial architectural parts of cell membranes from most prokaryotic and eukaryotic cells. In this paper, we use an array of biophysical techniques including differential scanning calorimetry, X-ray diffraction, infrared spectroscopy, atomic force microscopy, and molecular dynamics simulation to characterize the location and effects of TMCG on the phase transition and structural characteristics of phosphatidylethanolamine bilayers. We believe that the results presented here would help to clarify the molecular effect of TMCG on lipid bilayers, with the aim that this understanding will contribute to elucidate the molecular mechanism of the catechins’ biological actions.

## 2. Results and Discussion

The effects of TMCG on membranes was studied using model lipid membranes composed by dimiristoylphosphatidylethanolamine (DMPE). We used DSC to characterize the influence of the catechin on the thermotropic transition of DMPE and used X-ray diffraction, infrared spectroscopy, and atomic force microscopy to study the effect of TMCG on the structure and organization of the phospholipid. Finally, molecular dynamics simulation was used to determine the location and interaction of TMCG with the phosphatidylethanolamine bilayer.

### 2.1. DSC

**Figure 2:**
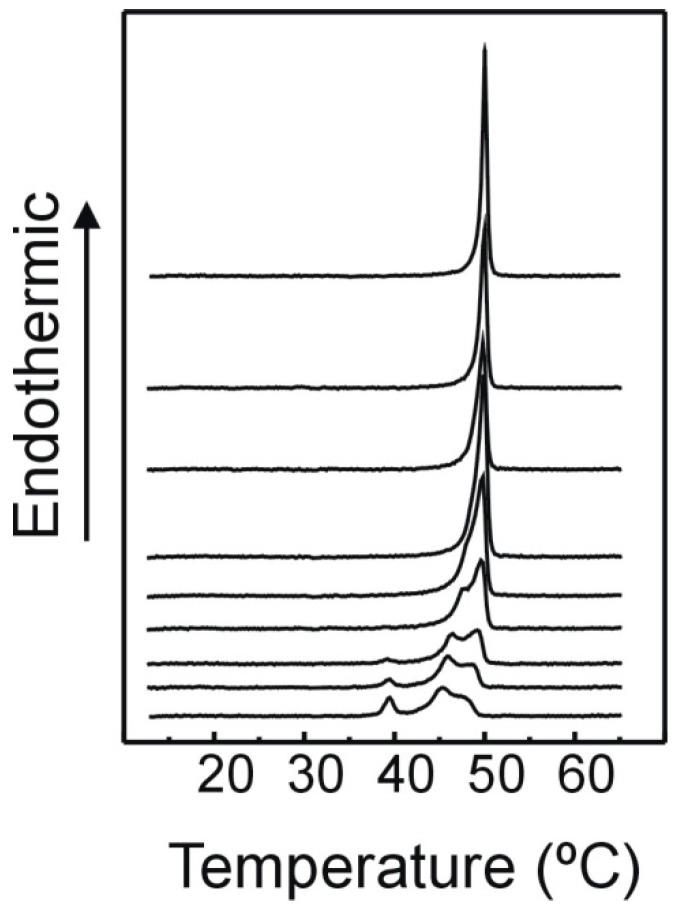
DSC thermograms depicting the effect of TMCG on the gel/liquid-crystalline phase transition of DMPE. Molar fraction of TMCG from top to bottom: 0, 0.02, 0.05, 0.07, 0.10, 0.15, 0.20, 0.25, and 0.30.

In Figure 2, the influence of TMCG on the thermotropic gel to fluid phase transition of DMPE is shown. Phosphatidylethanolamines containing saturated acyl chains exhibit transitions which are rather energetic and cooperative. The endotherm corresponds to the melting of the acyl chains from the L_β_ gel phase to the L_α_ liquid crystalline or fluid phase. When TMCG is not present, pure DMPE shows a transition temperature (Tc) at 49 °C and an enthalpy change of 6 kcal/mol, in accordance with earlier findings [29,30]. The presence of TMCG in DMPE system broadens the endotherm and shifts the transition to lower temperatures. Clearly, two broad endotherms are present at high concentrations of TMCG. In addition, at a TMCG molar ratio of 0.20, a highly cooperative endotherm appears with an invariable transition temperature of 37.7 °C. When the concentration of TMCG is further increased, this lower endotherm becomes more apparent. These results suggest that there is an interaction between TMCG and the phospholipid acyl chains which affects their structure, and doing so makes the transition less cooperative and lowers the temperature of the transition. The appearance of two broad components in the thermograms when TMCG is present at intermediate concentrations can be interpreted by the formation of domains which are rich in TMCG. In addition, the apparition of a lower temperature cooperative endotherm at higher TMCG concentrations suggests the formation of a stable complex or a different phase. The formation of a stable complex has been previously described for epicatechingallate in dimiristoylphosphatidylcholine [27] and for TMCG in dipalmitoylphosphatidylcholine membranes [28].

### 2.2. X-ray Diffraction

**Figure 3:**
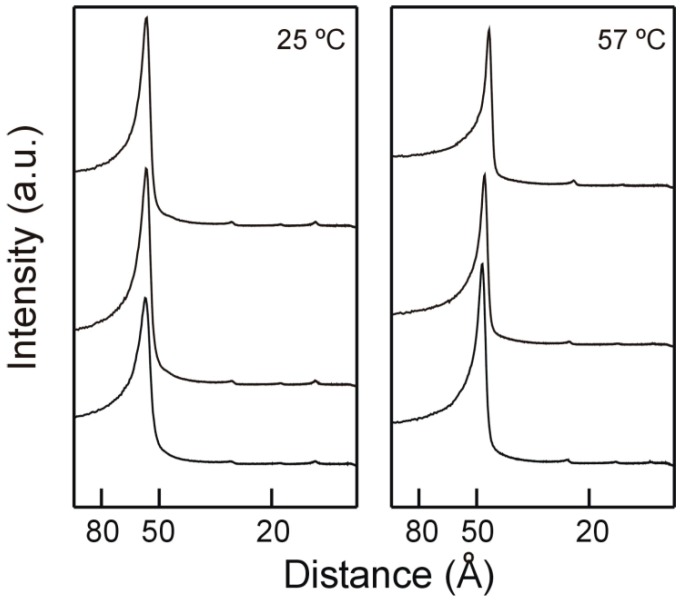
Small angle X-ray diffraction profiles of pure DMPE and mixtures with TMCG. Gel phase (**left**, 25 °C) and liquid crystalline phase (**right**, 57 °C). From top to bottom: pure DMPE, DMPE containing 0.07 mol fraction TMCG, DMPE containing 0.25 mol fraction TMCG.

We examined the structural characteristics of DMPE/TMCG system using Small Angle X-ray Diffraction (SAXD). Phospholipids which are organized in multilamellar structures produce reflections with distances relating as 1:1/2:1/3:1/4… [31]. Figure 3 presents the SAXD diffraction patterns corresponding to DMPE in the absence and in the presence of TMCG, in the gel and in the liquid crystalline phases. In the absence of TMCG, DMPE yields four reflections with distances which relate as 1:1/2:1/3:1/4, congruent to its anticipated multilamellar organization. The biggest first order reflection coincides with the intelamellar repeat distance (d-value), this value containing the thickness of the bilayer and the thickness of the interbilayer layer of water. A d-value of 56.1 Å is found when DMPE is in the gel phase (25 °C, Figure 3), whereas when DMPE reaches the liquid crystalline phase d-value decreases to approx. 47.7 Å (57 °C, Figure 3). These results agree with previous reports [30,32]. Three or four reflections relating as1:1/2:1/3:1/4 were found when TMCG is present in the system, supporting that the inclusion of the catechin does not change the lamellar architecture of the phospholipids. The presence of TMCG produces a small decrease in the interlamellar repeat distance of DMPE reaching 55.2 Å and 45.1 Å for the 0.25 molar fraction sample respectively below and above the transition temperature. An apparent membrane thinning effect of some catechins has been described before [26,33] and recently for TMCG present in DPPC systems [28].

**Figure 4:**
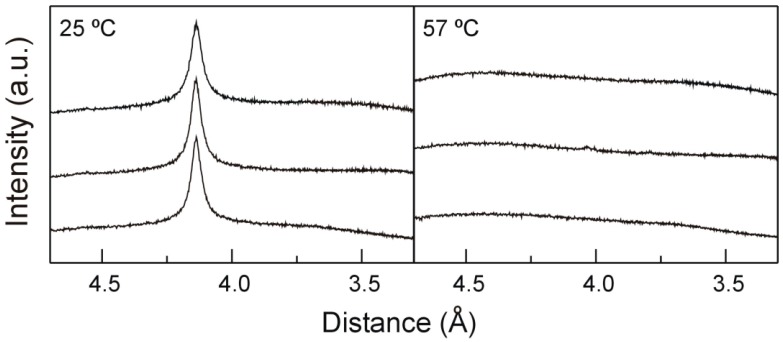
Wide angle X-ray diffraction profiles of pure DMPE and mixtures with TMCG. Gel phase (**left**, 25 °C) and liquid crystalline phase (**right**, 57 °C). From top to bottom: pure DMPE, DMPE containing 0.07 mol fraction TMCG, DMPE containing 0.25 mol fraction TMCG.

The packing symmetry of the phosphatidylethanolamine acyl chains can be deduced using X-ray diffraction in the wide angle region (WAXD, Figure 4). Before the transition takes place and in the absence of TMCG, DMPE displays a sole reflection at 4.14 Å which is sharp and symmetric (Figure 4, left) and is indicative of a hexagonal lattice packing of the acyl chains with the chains perpendicular to the membrane surface. This reflection is characteristic of the untilted L_β_ gel phase. After the transition, pure DMPE displays a broad diffuse reflection distinctive of the liquid crystalline L_α_ phase [34,35]. The inclusion of TMCG in DMPE systems does not alter this WAXD arrangement, indicating that TMCG—although it is capable of perturbing the structure of the phosphatidylethanolamine, making it more prone to undergo the thermotropic transition—is not able to perturb the hexagonal packing symmetry of the phosphatidylethanolamine while in the gel L_β_ phase.

We have previously found that, in dipalmitoylphosphatidylcholine systems, the complex formed at low temperatures in the presence of a high TMCG molar ratio had the characteristics of an interdigitated phase [28]. At difference with phosphatidylcholine, we show here that the presence of a high concentration of TMCG in phosphatidylethanolamine—though forming a complex at low temperatures—has the characteristics of a typical gel phase. This finding is in line with the known reluctance of phosphatidylethanolamine to interdigitate [36]. Phosphatitylethanolamines are not sensitive to form interdigitated phases in the presence of alcohol [37] or to form barotropic interdigitated phases [38]. It has been argued that the best explanation for this is the ability of the phosphatidylethanolamine head group to engage in hydrogen bonding [39]. On the other hand, phosphatidylcholine head groups are only capable of interaction through weaker electrostatic attractions [39]. Furthermore, the small dimension of phosphatidylethanolamine head group also permits a more compact interaction with less repulsion [40].

**Figure 5:**
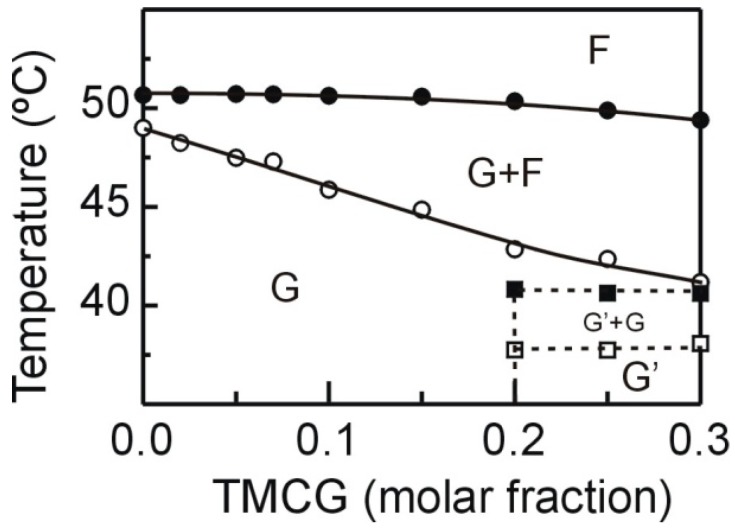
Partial phase diagrams for DMPE in DMPE/TMCG mixtures. Open and solid circles were obtained from the onset and completion temperatures of the gel to liquid crystalline phase transition. The phase designations are as follows: G, gel phase; F, liquid crystalline phase; and G′, immiscible gel phase.

With the information obtained from DSC and X-ray diffraction we made partial phase diagrams for the DMPE component in combination with TMCG. Figure 5 shows that, in our system, the solid and fluid lines exhibit almost ideal behavior, when the concentration of TMCG is incremented the temperature of both lines decreased, denoting good miscibility in the gel phase and also in the fluid one. The system proceeds from the gel phase (G phase) to the fluid (F phase) by means of a coexistence region (G + F) which is broader when TMCG is present at higher concentrations. At molar fractions higher than 0.20, the solid line remains nearly horizontal, indicating the existence of an immiscibility in the gel phase and evidencing the presence of a complex in a different gel phase (G′). The system evolving from the complex in gel phase (G′) to a liquid crystalline phase (F phase) through two regions of phase coexistence (G′ + G and G + F).

### 2.3. FT-IR

**Figure 6:**
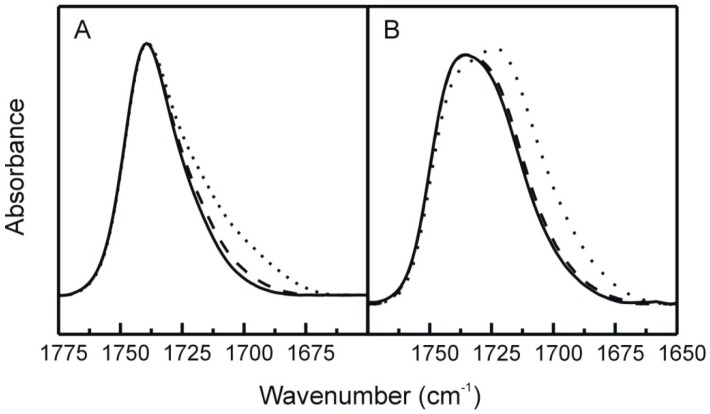
FT-IR ester carbonyl absorption band. Pure DMPE (solid lines), DMPE containing TMCG at 0.07 (dashed lines) and 0.25 (dotted lines) molar fraction, below (25 °C, panel A) and above (57 °C, panel B) the gel to fluid phase transition.

We used infrared spectroscopy to investigate the effects of TMCG on the interfacial region of DMPE bilayers. The infrared absorption band corresponding to the interfacial carbonyl groups of phosphatidylethanolamines experiences alterations when the phospholipid undergoes the transition from the gel phase to the fluid phase [41]. When TMCG is present in DMPE systems this carbonyl band of the infrared spectra of the phospholipid shows intriguing changes. We present in Figure 6 the shape of the carbonyl absorption band of DMPE. The band is moderately broad suggesting the presence of several components. It has been previously shown that the shape of the carbonyl band is formed by the combination of at least three different component centered at 1742, 1728, and 1714 cm^−1^ [29]. The transition from the gel to the fluid phase is accompanied by a clear increment in the low frequency part of the absorption band (Figure 6B) and it has been suggested that this change is due to the progression of the components centered at 1728 and 1714 cm^−1^ at the cost of the component centered at 1742 cm^−1^ [25]. These differences in the shape of the carbonyl band are significant as they account for the alteration in the hydrogen bonds that takes place at the water interface when the phospholipids experience the transition to the fluid phase. As it has been indicated previously, the components centered at 1728 and 1714 cm^−1^ correspond to hydrated carbonyl groups (hydrogen-bonded) while that centered at 1742 cm^−1^ corresponds to dehydrated carbonyl groups (non-hydrogen-bonded) [29,42]. Figure 6 also shows that when TMCG is present in DMPE system, there is a move of the maximum of the carbonyl band towards lower frequencies, presumably reproducing the rise in intensity of the components centered at 1728 and 1714 cm^−1^.

**Figure 7:**
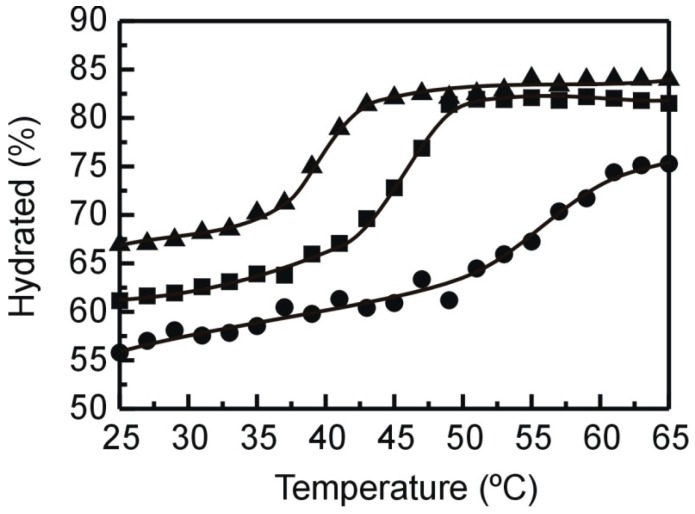
Proportion of the hydrated component of the carbonyl stretching band as a function of temperature. Pure DMPE (●) and mixtures containing TMCG at 0.07 (■) and 0.25 (▲) molar fraction.

We used curve fitting procedures to fit the spectra obtained from pure DMPE and mixtures with TMCG to three components centered at 1742, 1728, and 1714 cm^−1^. Gaussian-Lorentzian functions were used and we determined the areas corresponding to the different components. We present in Figure 7 the proportion of the population of hydrated carbonyls (the sum of components at 1728 and 1714 cm^−1^) versus temperature for the different mixtures. For pure DMPE, the percentage of hydrated carbonyls present in the gel phase (near 55%) is lower than that found in the fluid phase (near 75%). Previous studies indicated that this difference corresponds to the higher number of hydrogen bonds established by the carbonyl groups in the liquid crystalline phase accounting for the increase in hydration of the water interface which occurs in this phase [29]. The transition temperature is shifted to lower values in the presence of TMCG, which agrees well with the DSC results commented above. TMCG produces a greater number of hydrated carbonyls in the whole range of temperature, and this indicates that TMCG alters the water interface producing a higher number of hydrogen bonds between the phospholipid carbonyls and presumably the hydroxyl groups of the catechin derivative. Comparably higher numbers of hydrated carbonyls in the presence of TMCG have been recently described in phosphatidylcholine systems [28], which is in line with the increase in the degree of hydration of the phosphate group of phosphatidylcholine described previously for some catechins [27].

### 2.4. AFM

**Figure 8:**
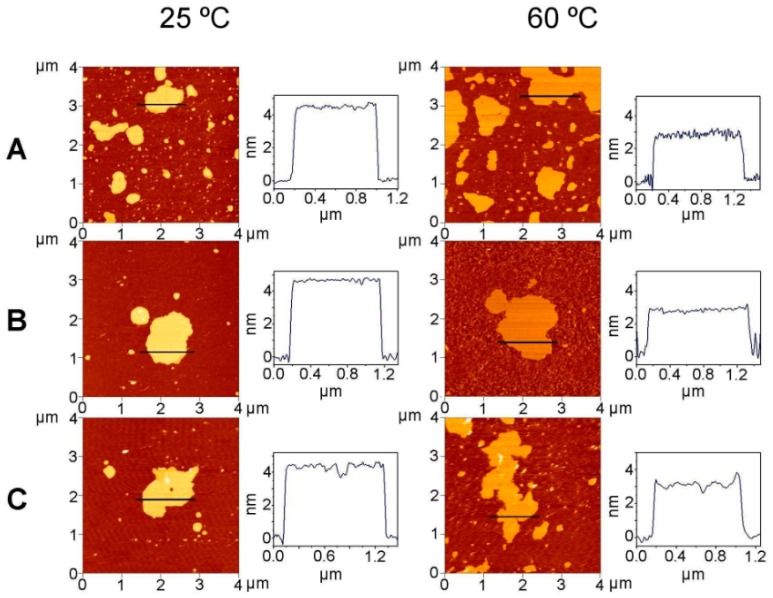
Topology of supported bilayers of DMPE systems containing different concentrations of TMCG as visualized by AFM. Pure DMPE (**A**); DMPE/TMCG 0.07 mol fraction (**B**); and DMPE/TMCG 0.25 mol fraction (**C**). The samples were scanned at 25 °C (gel phase) and 60 °C (liquid crystalline phase). Plots on the right correspond to the horizontal cross-sections at the indicated positions in the panels.

To check the topology of DMPE/TMCG systems, supported bilayers were studied through the use of AFM (Figure 8). Figure 8A contains images of multiple pure DMPE patches on a flat mica surface. The horizontal line profiles show that at 25 °C (gel phase) the pure DMPE bilayer thickness was 4.5 ± 0.1 nm, this value being close to the 4.7 nm value reported by Nussio and Voelker [43]. The measured thickness for pure DMPE in the liquid crystalline phase was 2.9 ± 0.2 nm, this distance is probably underestimated as it has been suggested that, in this phase, the scanning probe might deform and/or penetrate partially into the layer of fluid lipids [44]. When TMCG was present at 0.07 molar fraction (Figure 8B) the bilayer thickness was 4.7 ± 0.1 nm in the gel phase and 2.9 ± 0.1 nm in fluid phase. In the presence of TMCG 0.25 molar fraction (Figure 8C), the bilayer thickness was 4.4 ± 0.2 nm and 3.1 ± 0.2 nm, respectively. We could not see a clear effect of TMCG on the bilayer thickness of DMPE by AFM, however from the horizontal line profiles we can observe that in the presence of a high TMCG concentration (Figure 8C) the profiles were more irregular presenting clear depressions which might correspond with TMCG enriched domains. Given the scarce data available for phosphatidylethanolamines in the AFM literature, it should be emphasized that the DMPE systems shown in Figure 8 correspond to the same patches at different temperatures.

### 2.5. Molecular Dynamics Simulations

**Figure 9:**
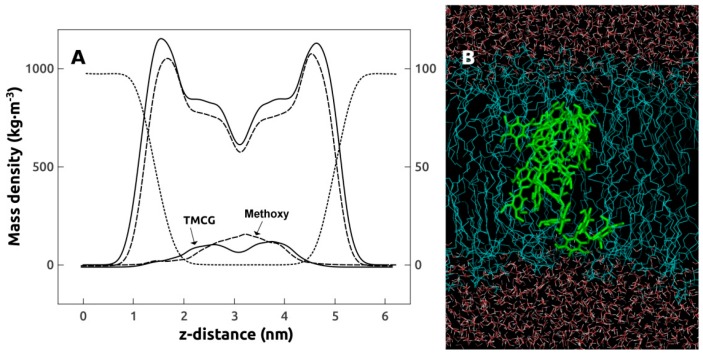
Molecular dynamics simulation on the interaction between TMCG and DMPE bilayers. (Panel **A**) Mass density profiles of water (dotted); DMPE bilayer in the absence (solid) and presence of TMCG (dashed); TMCG (solid, arrowed); and metoxy groups of TMCG (dashed, arrowed). All density profiles are scaled according to the left axis except that of metoxy groups of TMCG which are amplified (≈×10) and scaled to the right axis; (Panel **B**) Snapshot of the DMPE bilayer containing TMCG. The system under study is periodic in all directions, that is, the aqueous phase is continuous and TMCG can interact with both bilayer surfaces. Blue lines correspond to DMPE molecules and light-green lines to TMCG molecules. Red dots correspond to water molecules.

Molecular dynamics simulations were carried out to study the location of TMCG in the DMPE bilayer. We first analyzed the density profiles of the different components of the system along the *z*-axis which is normal to the DMPE bilayer. TMCG was originally situated in the water phase and, during the simulation, TMCG was able to interact with the bilayer. The precise location of the different components is presented in Figure 9A, in this picture it can be seen that the TMCG molecule is located mainly in the interior of the DMPE bilayer, although some portion of the molecule can interact with the water interface. The methoxy groups are the part of the TMCG molecule which locate deepest into the bilayer, emphasizing the importance of this chemical modification for the interaction of the drug with the membrane. The head group to head group thickness for DMPE in the absence of TMCG was 3 nm, this value decreasing to 2.85 nm in the presence of the catechin in agreement with the slight thinning effect in the interbilayer repeat distance determined by SAXD. To get a general view of how TMCG is distributed in the membrane, a selected snapshot of the TMCG/DMPE systems is shown in Figure 9B as representative picture. We determined the number of hydrogen bonds established by the carbonyl groups of DMPE and we found a 14 percent increase in these bonds in the presence of TMCG, this being in agreement with the increase in carbonyl groups hydration found by FT-IR. Molecular dynamics calculations shows that TMCG prefers to locate in the interior of DMPE bilayers and in this way it may perturb the phospholipid palisade and affect the thermotropic phase transition of the phospholipid, slightly altering the thickness of the membrane and increasing the hydrogen bonding pattern of the interfacial region.

Theses finding might be important to understand the mechanism for the activation or TMCG prodrug in melanoma [45], in this sense TMCG will have a strong affinity for the bilayer membrane, which is important given the membrane location of human tyrosinase. In addition, the findings presented in this paper, in relation to the interaction between TMCG and phosphatidylethanolamine membranes, will contribute to understand the role of natural chatechins as bioactive agents and increase the potential use of this synthetic derivative in other health-promoting actions of natural catechins.

## 3. Materials and Methods

### 3.1. Materials

Dimyristoylphosphatidylethanolamine (DMPE) was obtained from Avanti Polar Lipids Inc. (Birmingham, AL, USA). (−)-Catechin and 3,4,5-trimethoxybenzoyl chloride were from Sigma Chemical Co., Madrid, Spain). Purified water was deionized in a Milli-Q equipment from Millipore (Bedford, MA, USA), and filtered through 0.24 μm filters prior to use. All other reagents were of the highest purity available. The synthesis of TMCG was achieved from the commercially available catechin, following a process already described [17]. Solutions of DMPE were stored in chloroform/methanol (1:1) at −20 °C. The concentrations of phospholipids were calculated by phosphorous analysis [46].

### 3.2. Differential Scanning Calorimetry (DSC)

Convenient chloroform/methanol (1:1) solutions of DMPE and TMCG were mixed and the solvent was evaporated to dryness (nitrogen stream and 3 h at high vacuum). After the addition of 2 mL of buffer (100 mM NaCl, 0.1 mM EDTA, 10 mM Hepes pH 7.4) vesicles were formed by vortexing at temperature well above the gel to fluid transition temperature of the dehydrated DMPE. Samples containing 1 mg·mL^−1^ phospholpid were studied using a MicroCalMC2 calorimeter (MicroCal, Northampton, MA, USA) at a heating rate of 60 °C·h^−1^. The onset and completion temperatures obtained from the heating scans were used to construct the partial temperature-composition phase diagram.

### 3.3. X-Ray Diffraction

We used Nickel-filtered Cu Kα X-rays generated by a Philips PW3830 X-ray Generator operating at 50 kV and 30 mA, and a modified Kratky camera (MBraum-Graz-Optical Systems, Graz, Austria) equipped with two coupled linear position sensitive detectors (PSD, MBraum, Garching, Germany) monitoring the s-ranges (s = 2 sinθ/λ, 2θ = scattering angle, λ = 1.54 Å) between 0.0075–0.7 Å^−1^ and 0.20–0.29 Å^−1^ respectively as already defined [47]. We centrifuged (13,000 rpm) multilamellar vesicles containing 15 mg DMPE and the proper amount of TMCG and the obtained sediments were measured in a steel holder in contact with a Peltier unit, using cellophane windows. The samples were measured for 10 min with additional 10 min of temperature equilibration. For the purpose of determining the interlamellar repeat distances, d-spacings (d = λ/2sinθ) were plotted.

### 3.4. Infrared Spectroscopy (FT-IR)

Samples containing 15 mg DMPE and the proper amount of TMCG were prepared as described above in D_2_O buffer and arranged between CaF_2_ windows (25 mm × 2 mm) using 25 μm Teflon spacers and a Symta cell mount. We used a Nicolet 6700 Fourier-transform infrared spectrometer (FT-IR) (Madison, WI, USA). 256 interferograms with a nominal resolution of 2 cm^−1^ were collected every 2 °C with 5 min temperature equilibration employing a Peltier device (Proteus system from Nicolet, Madison, WI, USA). The subtraction of buffer spectra was carried out using Omnic or Grams software.

### 3.5. Atomic Force Micorsocopy (AFM)

Dried DMPE lipids (with and without TMCG) were resuspended in 100 μL buffer solution and vesicles were made by vortexing at 65 °C. Around 200 nm diameter liposomes were formed by mechanical extrusion at 60 °C through a Whatman nuclepore Track-Etched polycarbonate membrane (GE Healthcare Life Sciences, Barcelona, Spain) of this pore size. Liposomes were fused on recently cleaved mica by incubation at 65 °C, and excess was carefully rinsed with buffer afterwards. DMPE lipid bilayers were maintained at the desired temperature during AFM measurements using the JPK HCS (JPK Instruments AG, Berlin, Germany). JPK Nano Wizard II was used for AFM characterization. HQ:XSC11/Hard/Al BS silicon rectangular cantilever probes from Mikro Masch (Sofia, Bulgaria), with a typical force constant of 0.2 N/m and a typical resonant frequency of 15 kHz, were employed in dynamic mode. Scanning was always done in buffer solution, temperature was controlled by the JPK heating-cooling stage (HCS).

### 3.6. Molecular Dynamics Simulation

TMCG molecules were constructed as described previously [28], and DMPE topology was obtained from Piggot et al. [48]. The lipid bilayer was composed of two monolayers containing 36 DMPE molecules per monolayer, hydrated with a total of 2077 molecules of water and with or without four TMCG molecules randomly distributed in the water and lipid phases by using packmol software [49], yielding a 9:1 DMPE/catechin ratio. For water, the generic single point charge (SPC) water configuration [50] was used. The lipid bilayer was aligned such that it lied in the XY plane, i.e., the monolayers normal was parallel to the *z*-axis. Molecular dynamics calculations were carried out with GROMACS v4.5.4 molecular simulation package [51] under constant number of particles, pressure of 1 bar and temperature of 333 K, above the main phase transition temperature of DMPE. Gromacs 43A1-S3 force field, which is an improved force field for lipids based on GROMOS96 43a1, was used [52]. All other parameters were as previously described [28]. A total of 500 ns molecular dynamic simulations were carried out to allow relaxation and equilibration of the systems. The last 100 ns were collected for all calculations. PyMOL 1.5.0.1 [53] was employed to roughly inspect the arrangement of the catechin derivatives molecules in the lipid matrix and water phase and to capture images throughout the corresponding trajectories.

## 4. Conclusions

There is a large body of evidence which supports the view that phytochemicals, particularly catechins, exhibit great variety of biological functions which are beneficial to human health [54]. In this work we have studied the interactions of an antitumoral synthetic catechin derivative TMCG with dimistoylphosphatidylethanolamine membranes. The DSC data indicated that TMCG is capable of integrating into phosphatidylethanolamine bilayers and to interact with the phospholipids molecules, where it is able to shift the transition temperature of the gel to liquid-crystalline phase transition to lower values. TMCG is able to form enriched domains and, at higher concentrations, a new gel phase domain is found. X-ray diffraction experiments suggested that TMCG did not alter the overall bilayer organization of phosphatidylethanolamine, with only a minor decrease of the interlamellar repeat distance in the presence of TMCG. Infrared results evinced that TMCG increased the hydrogen bonding of the carbonyl interfacial group of the phospholipid. Horizontal line profiles obtained by AFM showed more irregularities in the presence of TMCG than in the pure phospholipid. The study by molecular dynamics simulation showed that TMCG is located in the interior of the phosphatidylethanolamine bilayer with access to the interfacial region of the membrane. All these results point to TMCG as a compound that interacts intimately with the phosphatidylethanolamine component of the membrane.

The ratios between TMCG and phosphatidylethanolamine used in this study are similar to those commonly used in previous studies on the interaction between catechins and membranes [26,27] and ranged from low TMCG concentration in the membrane (0.02 molar fraction) to high concentration of TMCG in the membrane (0.30 molar fraction). The correlation between these ratios and the concentrations of TMCG-exhibiting antimelanoma activity is not direct, but an approximation can be made. Data on the total amount of phospholipids per cell are very scarce. It has been recently reported for an epithelial cell line that the phospholipid content is around 2 μg Pi/10^6^ cells [55]. If we assume that the phospholipid content in melanoma cells is similar to this value and we consider the IC_50_ of 1.5 μM for TMCG in melanoma cells [17], it renders that under the cell culture conditions (number of cells and volume of the medium) the TMCG/Phospholipid ratio in the antiproliferative studies was around 0.3 molar fraction. Hence, the molar fraction used in our study is in the range of those expressing biological activity.

TMCG is a synthetically modified catechin and no data is yet available concerning physiological concentrations. In general, the issue of bioavailabilty of catechins is a very important one which needs to be addressed. In this sense, we believe that low concentrations of catechins in the blood stream would correspond to a much greater availability in the membrane fraction and they may accumulate over time to produce cellular concentrations that are much higher than that observed in serum samples. In addition, we would like to point out that the molar ratios studied in our model system are not necessarily required to be homogeneous in the whole cellular membrane. It would be enough that this TMCG/phospholipid ratio be attained locally in certain parts of the membrane. In this respect, the described propensity of TMCG to form enriched domains in the bilayer may help to locally attain higher concentrations of the molecule where it is needed.

The incorporation of TMCG into membranes is mainly driven by its lipophilicity, and this incorporation is the first step in the sequence of events induced by this compound. We have shown that this molecule is located in the interior of the bilayer, this location being crucial to reach tyrosinase, a membrane protein which acting on TMCG produces an activated metabolite able to inhibit dihydrofolate reductase and thus explaining the antimelanoma effect of TMCG [17]. In addition, we have shown that the presence of TMCG alters the structural properties of phospholipids and this would be also important from the point of view of other potential functional properties of this compound. The alteration of lipid bilayer properties may also affect the activity of integral proteins and hence influence other membrane-related processes. In fact, membrane proteins are solvated by the lipid bilayer, and the proteins and their host bilayer are energetically coupled through hydrophobic interactions. When membrane proteins undergo conformational transitions that involve their transmembrane domains, the bilayer adapts accordingly, and this bilayer adaptation incurs an energetic cost, which varies with changes in the bilayer physical properties [56]. In this sense, the changes that we have reported related to thickness, interfacial interactions, and formation of domains, may couple to changes in membrane protein function and might provide a mechanism by which TMCG could alter the function of diverse membrane proteins.

Given the complexity of biological membranes, the investigation of the structural parameters of phospholipids presented in this work is not possible to be performed using native biological membranes. The presence or different classes of phospholipids, sterols, and also proteins makes the use of physical techniques impractical to study these properties of phospholipids. We used model membrane systems as valuable tools to study the properties of individual phospholipid species. Many more studies concerning the interaction between TMCG and anionic phospholipids, the presence of mixtures of phospholipids with different head groups, or the influence of the presence of cholesterol are needed to obtain a clearer picture of the interaction of TMCG and membranes.

The compound under study was synthesized in order to increase the bioavalilabity of catechins and their ability to incorporate into cellular membranes and thus it differs from natural catechins in possessing a much more lipophilic gallic acid trimethylether moiety besides the polar phenolic groups at the catechin core. Thereby, a more pronounced amphiphilic character is induced which is likely to allow interactions with biological lipid bilayers in a more efficient way than would be expected for the natural congeners. Therefore, the results presented here should, at present, not be extrapolated to natural catechins such as those found, e.g., in green tea and other herbal preparations. Further studies will be required to investigate whether such membrane effects can also play a role in the bioactivity of these natural products.
